# Prospective evaluation of plasma pTau_217_ stability for the detection of Alzheimer’s disease in a tertiary memory clinic

**DOI:** 10.1186/s13195-025-01779-7

**Published:** 2025-07-05

**Authors:** Javier Arranz, Rosa Ferrer, Nuole Zhu, Sara Rubio-Guerra, Íñigo Rodríguez-Baz, José Enrique Arriola-Infante, Lucía Maure-Blesa, Jesús Garcia-Castro, Judit Selma-González, María Carmona-Iragui, Isabel Barroeta, Ignacio Illán-Gala, Miguel Santos-Santos, Juan Fortea, Alberto Lleó, Mireia Tondo, Daniel Alcolea

**Affiliations:** 1 Sant Pau Memory Unit, Department of Neurology, IR SANT PAU, Hospital de La Santa Creu I Sant Pau, Barcelona, Spain; 2 Barcelona Down Medical Center, Fundació Catalana Síndrome de Down, Barcelona, Spain; 3 Universitat Autònoma de Barcelona, Barcelona, Spain; 4 Servei de Bioquímica, IR SANT PAU, Hospital de La Santa Creu I Sant Pau, Universitat Autònoma de Barcelona, Barcelona, Spain; 5 Centro de Investigación Biomédica en Red en Enfermedades Neurodegenerativas (CIBERNED), Madrid, Spain; 6 Department of Neurology, Torrecárdenas University Hospital, Almería, Spain; 7 Centro de Investigación Biomédica en Red en Diabetes y Enfermedades Metabólicas (CIBERDEM), Madrid, Spain

**Keywords:** Stability, Plasma, Biomarkers, Alzheimer, Blood, Amyloid, Tau

## Abstract

**Background:**

Knowledge on the effect of analytical variability and storage conditions are essential for the successful implementation of plasma pTau_217_ in prospective settings.

**Aims:**

To investigate the performance of plasma pTau_217_, measured in consecutive samples with LUMIPULSE, for detecting Alzheimer’s disease in a prospective memory clinic setting, along with evaluating its pre-analytical and analytical stability.

**Methods:**

We prospectively measured pTau_217_ using the LUMIPULSE automated platform in consecutive patient plasma samples collected between May and November 2024 at the Sant Pau Memory Unit (Barcelona). A subset of participants also underwent paired lumbar puncture for CSF AD biomarkers. We compared biomarker concentrations under different short-term storage conditions (4ºC vs -20ºC) and different protocol pipelines, and assessed lot-to-lot variability. In the subset with available CSF biomarkers, logistic regression was used to evaluate the association between plasma pTau217 and the likelihood of a positive (A +) or a negative (A-) CSF amyloid status. Using ROC analysis, in this prospective cohort we evaluated the accuracy of previously established thresholds derived from historical samples.

**Results:**

We included 280 participants, divided into two groups: those with (*n* = 109) and without CSF data (*n* = 171). Among the subset with CSF, 48% were A + , with a plasma pTau_217_ fold-change of 4.5 × compared to A-. We found no differences in plasma pTau_217_ concentrations between either short-term storage conditions. The overall coefficients of analytical variation ranged from 1.8% to 3.2%. Plasma pTau_217_ concentrations were slightly higher in paired samples of the clinical protocol. Following a two-threshold approach, the need of confirmatory tests (grey zones) after measuring plasma pTau_217_ ranged between 45.9% and 18.3% using our previously reported strict or lenient cutoffs (overall accuracy 96.6% and 92.1%, respectively).

**Conclusions:**

The robust stability and low lot-to-lot variability of plasma pTau_217_ measurement in an automated platform result in high diagnostic performance of this biomarker in the prospective evaluation of patients in a memory clinic setting. These findings support its implementation into clinical routine, offering clinicians an accessible biomarker for AD diagnosis.

**Supplementary Information:**

The online version contains supplementary material available at 10.1186/s13195-025-01779-7.

## Introduction

Early diagnosis of Alzheimer disease enables the timely initiation of social and therapeutic interventions, as well as the opportunity to receive disease-modifying treatments or participate in clinical trials. Gold-standard biomarkers such as Amyloid-PET and CSF are invasive or not widely available in routine clinical practice, whereas blood-based biomarkers provide a more cost-effective and accessible alternative [1–5]. Plasma pTau_217_ has demonstrated high accuracy in differentiating symptomatic Alzheimer’s disease (AD) from other neurodegenerative diseases [6, 7] and has positioned as one of the most promising plasma-based tau species [8–19]. Measurement of plasma pTau_217_ is feasible across various platforms [8, 10, 20–26] with the LUMIPULSE platform exhibiting particularly high accuracy in recent studies [27, 28]. Furthermore, as a fully automated system, this platform reduces the risk of analytical variability, thus facilitating safer translation into clinical practice [29–35].

However, the performance of this biomarker in real-world clinical settings remains poorly understood, where slight changes in extraction protocols, storage conditions or reagent variations could impact biomarker levels, thereby influencing its diagnostic performance [33, 34]. Therefore, assessing the stability of pTau_217_ under different real-world conditions is critical, as pre-analytical and analytical variations could lead to misdiagnosis [36–38]. Understanding its performance in routine blood draw scenarios, rather than controlled research laboratory environments, will help determine the impact of standard procedure variations on the biomarker's robustness and reliability.

In a previous study, we assessed the diagnostic performance of plasma pTau_217_ for the detection of amyloid pathology in CSF in a collection of stored samples using a two-threshold approach, finding an overall accuracy of over 90% in symptomatic groups, with the potential to reduce confirmatory testing by 50% to 80% [6]. In this study, we evaluated the performance of these cutoffs in a new, prospective real-world setting, using the routine blood collection pipeline of the hospital. Additionally, we examined the impact of different short-term storage conditions and batch-to-batch analytical variability on biomarker concentrations. These factors are essential for the successful implementation of this approach in routine laboratory workflows.

## Methods

### Study participants and clinical classification

We included consecutive patients assessed prospectively in the Sant Pau Memory Unit that underwent blood extraction as part of their diagnostic work-up [39] between May and November 2024. A subset of patients with a consistent clinical diagnosis of mild cognitive impairment (MCI) or early dementia and with no contraindications for the procedure underwent lumbar puncture for the analysis of AD CSF biomarkers and had matched CSF and blood samples collected. Patients in this subset, were further classified as A + or A − based on the CSF Aβ_1–42_/Aβ_1–40_ ratio [40].

The Sant Pau Memory Unit covers a population area of approximately 450.000 inhabitants, and patients are typically referred by primary care physicians or other departments in the hospital due to concerns in cognitive performance. Each year, we provide care to over 3,700 patients, including 700 first-time visits and 3,000 follow-up appointments.

At the time of CSF and/or plasma sample collection, participants had a syndromic clinical diagnosis of subjective cognitive impairment (SCI), mild cognitive impairment (MCI) or dementia. Clinical diagnoses were established following comprehensive neurological, neuroimaging and neuropsychological assessments [39]. Following a comprehensive evaluation that included analysis of AD biomarkers in CSF or plasma, participants were classified based on their etiologic diagnosis into one of the following categories: Alzheimer’s Disease (AD), Frontotemporal Lobar Degeneration (FTLD), Lewy Body Disease (LBD) or other non-neurodegenerative diseases (OND). A subset of participants received a classification of “Unknown,” as their etiologic diagnosis remained uncertain following the initial evaluation, requiring further clinical follow-up. To evaluate the impact of kidney dysfunction on biomarker concentrations, data on estimated glomerular filtration rate (eGFR) were collected.

### Sample collection and analysis

#### Blood and CSF protocols

Blood samples were collected in EDTA-K2 tubes under fasting conditions before 11 a.m. and then transferred to the clinical laboratory following the routine blood collection pipeline of the hospital. Upon arrival, samples were centrifuged (2000 g for 10 min at 4 °C), aliquoted in 5 mL polystyrene tubes (Falcon™, Ref. 352,052), and stored at −80 °C for subsequent analysis, all within a 4-h timeframe.

In the subset of patients that underwent lumbar puncture, blood was collected immediately after lumbar puncture simultaneously in two EDTA-K2 tubes. One tube (A) was sent to the clinical laboratory as described above, and a second tube (B) was sent to our research laboratory, where it was processed following our previously described research protocol [39]. Briefly, blood samples were centrifuged (2000 g for 10 min at 4 °C) within 2 h of collection, then plasma was aliquoted in 1.5 mL polypropylene tubes (Sarstedt, Ref. 72.690.001) and stored at −80 °C until analysis. This approach allowed a direct comparison between the clinical practice protocol (A) and our standardized research protocol (B). CSF samples were collected by lumbar puncture, then centrifuged, aliquoted in 1.5 mL polypropylene tubes (Sarstedt, Ref. 72.690.001) and stored at −80ºC until their analysis. The detailed protocol for CSF samples collection used at our facility has been previously documented [39].

Clinical plasma samples (A), pTau217 were measured prospectively every week in the fully automated Lumipulse G600II platform with commercially available kits from Fujirebio Europe (Ghent, Belgium) between May and November 2024, using different reagent lots. Plasma samples processed in the research laboratory (B) were analyzed in one batch in November 2024 using a consistent reagent lot. Plasma Aβ_1–42_/Aβ_1–40_ samples were measured only in participants with eGFR < 60 mL/min/m2 in the Lumipulse fully-automated platform G600II using commercially available kits (Fujirebio Europe, Ghent, Belgium). On the day of testing, plasma samples were brought to room temperature, thoroughly mixed, centrifuged at 2000 g for 5 min, and transferred to specific cuvettes for analysis on the Lumipulse platform.

CSF biomarkers, Aβ_1–42_, Aβ_1–40_, pTau181 and tTau, were measured as part of the validation process for blood biomarkers. These measurements were conducted during routine runs scheduled every two weeks following previously reported methods [40]. Based on CSF biomarker levels, participants were categorized as amyloid positive (A +, CSF Aβ_1–42_/Aβ_1–40_ < 0.062) or negative (A-). The validation of this cutoff value has been described in prior studies [40].

The study was approved by the Sant Pau Ethics Committee (Protocol code: EC/22/202/6880) following the standards for medical research in humans recommended by the Declaration of Helsinki. All participants or their legally authorized representative gave written informed consent to participate in biomarkers research studies.

### Stability of samples and batch-to-batch variability (fridge vs freezer; lot-to-lot variability)

**Fig. 1:**
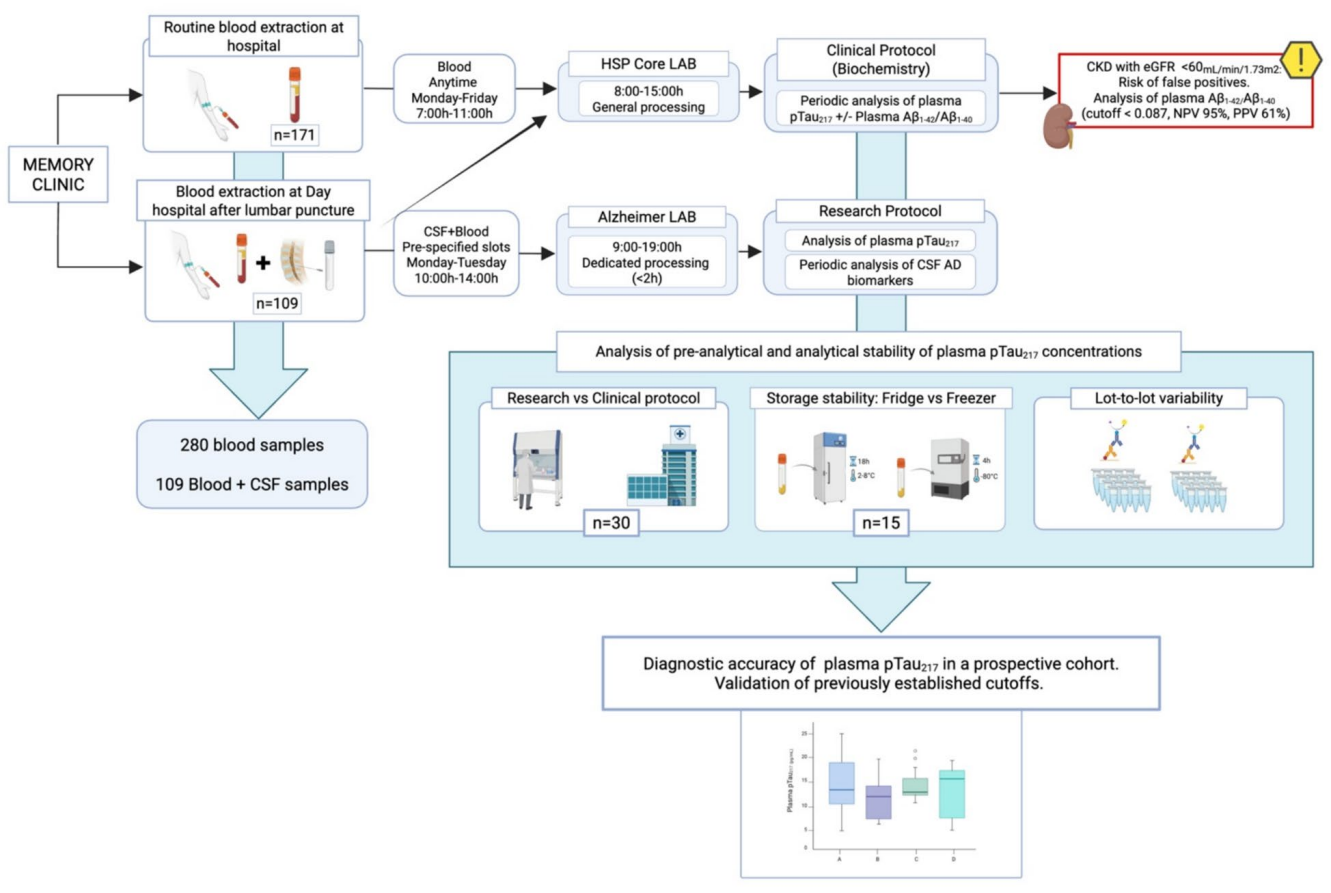
Flowchart of the implementation of plasma biomarkers in clinical routine. The figure illustrates the workflow for processing plasma and CSF samples in a real-world memory clinic setting. Patients with mild cognitive impairment (MCI) or mild-to-moderate dementia, who were clinically suspected of having Alzheimer’s disease (AD) but had contraindications for lumbar puncture (LP), underwent routine blood extraction at the hospital for plasma pTau_217_ measurement. These blood samples followed the standard hospital protocol, being sent to the core laboratory and subsequently to the biochemistry (protein) department for the analysis of AD plasma biomarkers. For patients with no contraindications for LP, who were diagnosed with MCI or mild dementia and were under 85 years of age, both LP and blood extraction were conducted at the Day Hospital. CSF and subsamples of plasma were sent to the Alzheimer Laboratory and processed according to the research protocol. Plasma samples were also sent to the core laboratory and subsequently analyzed for AD plasma biomarkers in the biochemistry department. Additionally, in a subset of participants with an eGFR < 60 mL/min/m.^2^, the Aβ_1–42_/Aβ_1–40_ ratio was analyzed to help mitigate the limited but potential impact of pTau_217_ false positives in this population. Plasma pTau_217_ concentrations from both processing protocols (clinical and research) were compared. Variability due to storage conditions and lot-to-lot differences was assessed. Finally, the accuracy of previously established cutoffs for plasma pTau_217_ was validated within this prospective cohort, and newly calculated cutoffs obtained from this prospective cohort were proposed. Created in BioRender. (2025) https://BioRender.com/x23u713

To evaluate the stability of the analysis, 15 consecutive blood samples collected in EDTA-K2 tubes were either processed within a 4-h timeframe in the clinical laboratory, as previously described, or stored overnight at 4 °C (range: 2–8 °C) before being processed the following morning, resulting in an 18-h delay. Additionally, since different production lots were used for the pTau_217_ analysis, we recorded lot references to assess potential lot-to-lot variability. Finally, potential differences between the clinical (A) and research (B) laboratory protocols were evaluated by analyzing 30 plasma samples processed simultaneously under both protocols, as described earlier. A descriptive flowchart of the study is shown in Fig. 1.

### Statistical analysis

Data normality was evaluated using the Shapiro–Wilk test. For continuous variables following a normal distribution, Student's t-test was employed. For those not normally distributed, Wilcoxon rank-sum tests or log-transformed linear regression were applied. ANCOVA was conducted for group comparisons. Chi-square test was used to assess differences in categorical variables, with Fisher’s exact test applied to group comparisons with smaller sample sizes.

Diagnostic accuracy of plasma pTau_217_ was evaluated through receiver operating characteristic (ROC) analysis. We calculated the areas under the curve (AUC) for plasma pTau_217_ and logistic regression models that combined plasma pTau_217_ with other variables. A basic model incorporating Age and Sex served as a reference to assess the added diagnostic value of plasma pTau_217_. We compared the accuracy of plasma pTau_217_ and regression models using DeLong's test adjusted for multiple comparisons via the Bonferroni method. Sensitivity, specificity, and Youden’s J index were calculated across a range of cutoffs to differentiate A + from A- participants within the cohort. Concordance of pTau_217_ with CSF amyloid status was analyzed. Using cutoffs derived from predictive models in retrospective research samples from our prior work [6], we stratified the participants into low, medium, and high risk of CSF amyloid pathology, applying both strict (97.5% sensitivity and specificity) and lenient (95% sensitivity and specificity) cutoffs, and compared their performance with newly calculated cutoffs derived from this prospective cohort. We evaluated the variations in cutoff performance (negative predictive value [NPV], positive predictive value [PPV], and global accuracy) across different clinical scenarios based on the expected prevalence of amyloid pathology. All analyses were performed using R statistical software (version 4.2.1), with an alpha level set at 0.05.

## Results

### Study participants

We included 280 participants with plasma measures. They were further divided in two groups according to whether CSF was also obtained or not (Table 1). Compared to participants who underwent lumbar puncture, participants without CSF were older (72 vs 74 years, *p*=0.008), had higher plasma pTau217 concentrations (0.18 vs 0.25 pg/mL, *p* = 0.036), were more likely to be in more advanced stages (42.4% were GDS ≥ 4 vs 19.4%, *p*<0.001) and had higher prevalence of moderate-severe kidney dysfunction defined as eGFR <60 mL/min/1.73m2 (11% vs 22%, *p*=0.001). There were no differences in sex distribution or years of education between both groups, and the distribution of clinical diagnosis was also comparable (*p*=0.4).

**Table 1:** Characteristics of patients with and without CSF

Characteristic	With CSF, *N* = 109^a^	Without CSF, *N* = 171^a^	*p*-value^2^
Age (years)	72 (7)	74 (8)	0.008
Sex (female)	62 (57%)	84 (49%)	0.2
Education (years)	11.1 (4.5)	12.1 (4.8)	0.082
Plasma pTau_217 (median[IQR])_	0.18 [0.10–0.46]	0.25 [0.13–0.54]	0.036
Plasma Aβ_1–42/_ Aβ_1–40 (median[IQR])_	0.070 [0.063—0.078]	0.074 [0.068—0.079]	0.4
Amyloid status			> 0.9
A-	57 (52%)	NA	
A +	52 (48%)	NA	
GDS			< 0.001
≤ 3	87 (81%)	95 (58%)	
≥ 4	21 (19%)	70 (42%)	
eGFR _(mL/min/1.73m2)_			0.001
> 90	33 (30%)	24 (14%)	
60–90	64 (59%)	110 (64%)	
< 60	12 (11%)	37 (22%)	
Clinical diagnosis			0.4
Alzheimer's Disease	44 (40%)	74 (43%)	
FTLD	14 (13%)	21 (12%)	
LBD	13 (12%)	28 (16%)	
OND	33 (30%)	45 (26%)	
Unknown	5 (4.6%)	2 (1.2%)	

^a^Unless otherwise specified, numeric variables are shown as mean(SD). Categorical variables are shown as percentage (%)

^2^ Wilcoxon rank sum test, Pearson’s chi-squared test, Wilcoxon rank sum exact test, fisher’s exact test

Abbreviations: pTau_217_ phosphorylated tau 217, Aβ_1–42_ Amyloid β_1–42_. Aβ_1–40_ Amyloid β_1–40_. GDS Global Deterioration Scale, eGFR estimated glomerular filtration rate, FTLD Frontotemporal Lobar Degeneration-related disorders, LBD Lewy Body Dementia, OND Other Non-neurodegenerative

Within the subset with CSF (Table 2), 57 were A- and 52 were A+. The A+ participants were older (70 vs 75 years, *p* < 0.001) and had higher plasma pTau217 concentrations (4.5 folds higher in A+, *p*<0.001.

**Table 2:** Characteristics of patients with CSF sample

Characteristic	A-, *N* = 57^a^	A +, *N* = 52^a^	*p*-value^2^
Age (years)	70 (7)	75 (5)	< 0.001
Sex (female)	28 (49%)	34 (65%)	0.087
Education (years)	11.3 (4.8)	10.9 (4.3)	0.6
Plasma pTau_217 (median[IQR])_	0.11 [0.08—0.15]	0.49 [0.24—0.74]	< 0.001
Plasma Aβ_1–42/_ Aβ_1–40 (median[IQR])_	0.080 [0.068—0.087]	0.063 [0.061—0.071]	0.006
GDS			0.047
≤ 3	50 (88%)	37 (73%)	
≥ 4	7 (12%)	14 (27%)	
eGFR (mL/min/1.73m2)			0.010
> 90	24 (42%)	9 (17%)	
60–90	26 (46%)	38 (73%)	
< 60	7 (12%)	5 (9.6%)	0.56
Clinical diagnosis			< 0.001
Alzheimer's Disease	1 (1.8%)	43 (83%)	
FTLD	12 (21%)	2 (3.8%)	
LBD	9 (16%)	4 (7.7%)	
OND	30 (53%)	3 (5.8%)	
Unknown	5 (8.8%)	0 (0%)	

^a^ Unless Otherwise Specified, Numeric Variables Are Shown As Mean(SD). Categorical variables are shown as percentage (%)

^2^Wilcoxon Rank sum test, Pearson’s Chi-squared test, Wilcoxon rank sum exact test, fisher’s exact test

Abbreviations: *pTau*_*217*_ phosphorylated tau 217, *Aβ*_*1–42*_ Amyloid β_1–42_. *Aβ*_1–*40*_ Amyloid β_1–40_. *GDS* Global Deterioration Scale, *eGFR* estimated glomerular filtration rate, *FTLD* Frontotemporal Lobar Degeneration-related disorders, *LBD* Lewy Body Dementia, *OND* Other Non-neurodegenerative

### Accuracy, PPV and NPV of historical cutoffs and AUC of pTau_217_ in the prospective clinical cohort

We evaluated in the prospective clinical cohort the accuracy of the cutoffs obtained in the retrospective cohort. According to our previously calculated two-threshold strict cutoffs (sensitivity 97.5% = 0.13 pg/mL, specificity 97.5% = 0.55 pg/mL), the proportion of participants classified as low, medium or high risk of amyloid pathology in the whole cohort were 34.9%, 45.9% and 19.3%, respectively. Applying more lenient cutoffs (sensitivity and specificity above 95%), the proportions were 54.1%, 18.3% and 27.5%, respectively.

**Fig. 2:**
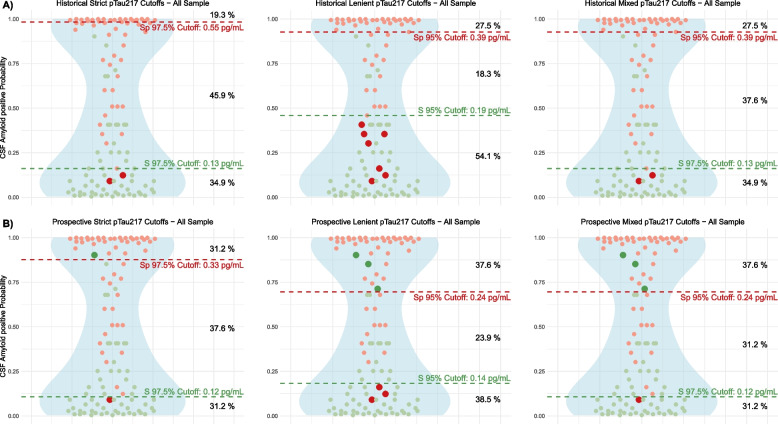
Application of previously established historical cutoffs (**A**) and newly calculated cutoffs (**B**) to the prospective cohort. The figure illustrates the application of previously established cutoffs to the validation cohort, represented by dashed red and green lines at distinct levels on the Y-axis, each corresponding to specific sensitivity and specificity values. Red dots denote individuals with amyloid positivity in cerebrospinal fluid (CSF), while green dots represent amyloid-negative cases. Participants with pTau_217_ concentrations above the dashed red line are classified as high risk for amyloid CSF positivity, those between the dashed red and green lines are classified as medium risk, and those below the dashed green line are categorized as low risk. Misclassifications into high or low-risk groups are indicated with distinct marker sizes and colors. On the right, the figure displays the percentages of the sample allocated to each risk category. pTau_217_: phosphorylated tau 217. S, sensitivity. Sp, specificity

In the subset with confirmatory CSF, we evaluated the accuracy of historical cutoffs to discriminate between A + and A- patients prospectively. The prevalence of A + was 48%. In our prospective evaluation, the global accuracy for historical strict cutoffs in this prospective setting was 96.6%, with NPV 95% and PPV of 100%. The global accuracy of historical lenient cutoffs was 92.1%, with a NPV of 88% and a PPV of 100%. Applying the strict cutoffs, only two observations were misclassified, both identified as A- by plasma pTau_217_ but as A + by CSF Aβ_1–42_/Aβ_1–40_: a male with a final clinical diagnosis of LBD and a female with clinical diagnosis of FTLD. Of note, both patients were CSF pTau_181_ negative. From the seven observations misclassified with lenient cutoffs, six were female, six were CSF pTau negative, and two had etiologic diagnosis of LBD, two of FTLD and three of AD. Using combined cutoffs (sensitivity 97.5% and specificity 95%), global accuracy was 97.03%, leaving 37.6% of the sample in the intermediate zone. The performance of the three historical combinations is shown in Fig. 2A.

### Cutoffs calculated in the prospective evaluation

The AUC for detecting A positivity obtained from the prospective cohort was 0.95 (95% CI 0.91–0.98) for plasma pTau_217_ (Supplementary Fig. 1). The models of age and sex or the combination of age, sex and plasma pTau_217_ did not outperform plasma pTau_217_ alone. The single cutoff that maximized the Youden index was 0.185 pg/mL, with a sensitivity of 86.5% and a specificity of 91.2%, NPV of 88.1% and PPV of 90%, with an accuracy of 89%.

Using a two-cutoff approach to stratify the risk of AD, we found no significant differences in the biomarker's overall accuracy compared to our retrospective cohort. However, the optimal strict and lenient PPV and NPV cutoffs varied. For strict cutoffs (97.5% sensitivity and specificity), the optimal NPV threshold was 0.12 pg/mL, closely matching the previous 0.13 pg/mL, while the optimal PPV threshold decreased to 0.33 pg/mL from 0.55 pg/mL. For lenient cutoffs (95% sensitivity and specificity), the optimal NPV threshold was 0.14 pg/mL, lower than the previous 0.19 pg/mL, and the PPV threshold decreased to 0.24 pg/mL from 0.39 pg/mL.

**Fig. 3:**
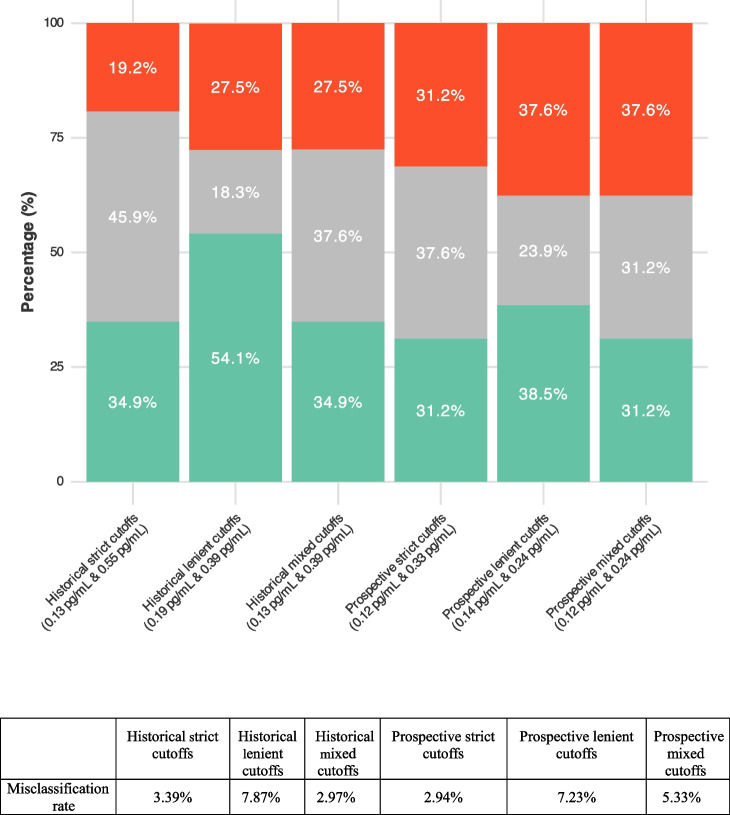
Risk stratification areas across different cutoff combinations. Stacked area chart of the distribution of the different risk areas across different two-threshold approaches in the subset with CSF. In the table we show the misclassification rate related to each approach. We can observe that lenient cutoffs reduce the grey zone with the tradeoff of higher misclassification rate

Applying the prospective cutoffs, the proportion of cases with medium risk was 37.6% using strict cutoffs, and 23.9% with lenient cutoffs. The global accuracy of the prospective cutoffs to detect CSF amyloid positivity was similar to that of previously reported cutoffs, as the misclassification rates were 2.94% for the strict cutoffs and 7.23% for the lenient combination, which were comparable to 3.39% and 7.87%, respectively, using the previous historical cutoffs. The accuracies of different cutoff combinations in our prospective assessment are shown in Table 3. The performance of the three prospective combinations is shown in Fig. 2B, and the proportion of participants classified in low, medium or high risk of having AD with the use of different cutoff combinations is shown in Fig. 3. Clinical and demographic details on the individuals in the grey zone are provided in Supplementary Text 1.

**Table 3:** Diagnostic performance of different cutoff combinations of plasma pTau_217_ to detect A + participants

Cutoffs	Values	Sensitivity	Specificity	NPV	PPV	Accuracy	Prevalence	Grey Zone
Historical strict cutoffs [6]	0.13—0.55	91.3	100	94.7	100	96.6	47.7	45.9
Historical lenient cutoffs [6]	0.19—0.39	81.1	100	88.1	100	92.1	47.7	18.3
Historical mixed cutoffs [6]	0.13—0.39	93.8	100	94.7	100	97.1	47.7	37.6
Prospective strict cutoffs	0.12—0.33	97.1	97.1	97.1	97.1	97.1	47.7	37.6
Prospective lenient cutoffs	0.14—0.24	92.7	92.9	92.7	92.9	92.8	47.7	23.9
Prospective mixed cutoffs	0.12–0.24	97.4	91.7	97.1	92.6	94.7	47.7	31.2

*pTau*_*217*_ phosphorylated tau 217, *NPV *Negative predictive value, *PPV* Positive predictive value. Threshold units for pTau_217_ are in pg/mL

### Variations in PPV, NPV, and global accuracy across different cutoff combinations in clinical scenarios with varying prevalence of amyloid pathology

In our prospective cohort, the prevalence of amyloid pathology was approximately 50%. We evaluated the impact of varying prevalence rates on accuracy and predictive values. Supplementary Fig. 2 illustrates the NPV increase and PPV decrease in settings with lower A + prevalence, such as primary care during cognitive screening. Conversely, in settings with higher A + prevalence, such as memory clinics, the PPV increases while the NPV declines. The global accuracy of plasma pTau_217_ was above 90% in the context of A + prevalences ranging from 20 to 60%.

### Performance of historical cutoffs in the subset without CSF

The risk stratification distribution in the subset without CSF, was largely comparable to that of patients with confirmatory CSF. With strict cutoffs, 24.6% are classified as pTau_217_ positive, 50.8% fall within the gray zone, and 24.6% are pTau_217_ negative. For lenient cutoffs, the distribution is 36.8% pTau_217_ positive, 22.8% in the gray zone, and 40.4% pTau_217_ negative—both showing a slight increase in the gray zone and an increase in positive cases compared to the CSF group.

### Effect of chronic kidney disease on plasma pTau_217_ accuracy

In this cohort, twelve participants with available samples of CSF (five A + and seven A-) had moderate or severe kidney dysfunction (eGFR < 60 mL/min/1.73m^2^). Using historical strict cutoffs (0.13 pg/mL—0.55 pg/mL), four of the five A + cases were correctly classified as high AD risk according to plasma pTau_217_, and the remaining case was classified as medium risk (pTau_217_ of 0.5 pg/mL). Interestingly, in this case, the Aβ_1–42_/Aβ_1–40_ ratio in plasma (0.067) was below the previously proposed cutoff for individuals with eGFR < 60 mL/min/1.73m^2^ (0.087). This observation remains consistent with our classification algorithm, which suggests that the Aβ_1–42_/Aβ_1–40_ ratio may serve as a valuable biomarker in cases of moderate to severe kidney dysfunction. The remaining seven A- cases were classified as either negative (two) or within the gray zone (five). We found differences in the prevalence of participants with eGFR < 60 mL/min/m2 in the group without CSF. This variable did significantly impact pTau_217_ concentrations after adjustment for age and GDS, which were the three variables identified as the primary contributors to differences in pTau_217_ concentrations between the groups.

### Pre-analytical variability

#### Short-term storage conditions

**Fig. 4:**
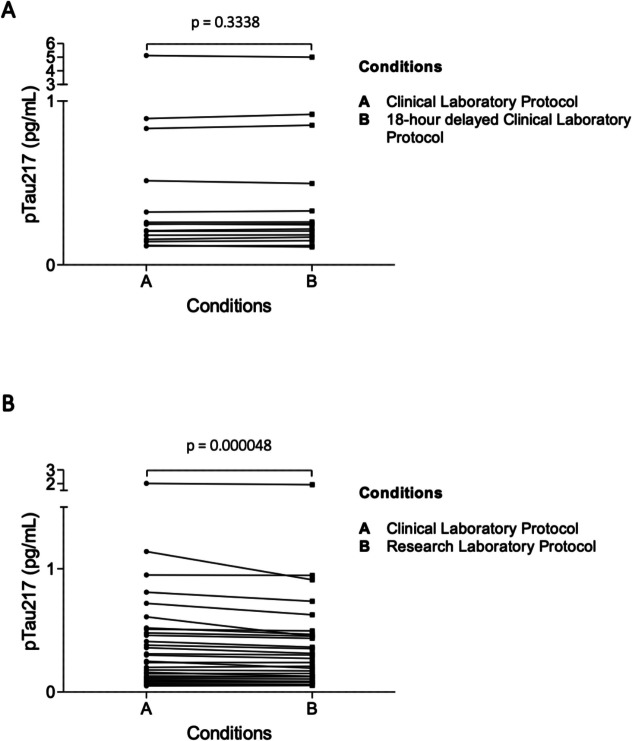
Variability associated to storage conditions (**A**) and comparison between Clinical and Research protocols (**B**). 4 A displays the results of paired Wilcoxon tests comparing clinical laboratory storage conditions (4 h vs. 18 h), revealing no significant differences between them (*p* = 0.3338). In contrast, **B** presents paired Wilcoxon tests comparing the clinical (**A**) and research (**B**) protocols, showing slightly higher pTau217 concentrations under the clinical protocol (*p* = 0.000048)

For the pTau_217_ stability study, 15 plasma samples were either frozen within a 4-h window in accordance with the clinical laboratory protocol or stored in the refrigerator (4ºC) for 18 h before freezing, introducing a storage delay. As shown in Fig. 4A, levels of pTau_217_ were not significantly different between both short-term storage conditions (*p* = 0.334). The mean coefficient of variation (CV) between both conditions was 2.19% and the mean percentual change was 1.09%.

### Clinical vs. research protocol

To evaluate potential differences between the clinical and research laboratory protocols, we compared plasma pTau_217_ concentrations of 30 plasma samples that were processed under both protocols as described above. As illustrated in Fig. 4B, pTau_217_ levels in Condition A did differ significantly from those in Condition B (p < 0.001), finding slightly higher concentrations in clinical protocol. The mean CV between both protocols was 6.3% and the mean percentual change was 7.07%.

### Analytical variability: lot-to-lot variability

To assess potential lot-to-lot variability, we calculated the inter-assay CV for plasma pTau_217_ across two control levels. Both control samples were included in 12 assay runs, with either the high or low control included in the remaining 14 runs. The overall CV across 26 different runs and three different lots of the pTau_217_ assay (#4097, #4129 and #5023) was 3.2% for the low control (0.49 pg/mL) and 1.8% for the high control (3.90 pg/mL). Lot-to-lot variability is presented in Supplementary Table 3.

## Discussion

In this study, we evaluated the diagnostic performance of plasma pTau_217_ in a real-world memory clinic prospective application. We found that the implementation of this analysis is feasible and that different combinations of cutoffs showed excellent diagnostic accuracy (92–96%), similar to that reported in the primary retrospective cohort. We also found that variations in the pre-analytical process, short-term storage conditions, or the use of different batches of analytical reagents had minimal impact on plasma pTau_217_ concentrations. We observed small but statistically significant differences in plasma pTau_217_ concentrations between clinical and research protocols, with minimal impact on biomarker performance. Importantly, the absence of differences between short-term storage conditions highlights its robustness, making it highly suitable for clinical laboratory use. Overall, the results support the potential of plasma pTau_217_ for integration into real-world clinical workflows and underscore the importance of evaluating such differences before implementation in routine clinical practice.

Previous studies have extensively evaluated the diagnostic performance of plasma pTau_217_ compared to CSF and PET, demonstrating its high accuracy in detecting amyloid pathology across different clinical stages. Our prior research identified plasma pTau_217_ as the most effective standalone biomarker to detect amyloid pathology in CSF, with an area under the curve (AUC) of 0.94, a performance that remained consistent across different cognitive stages. Our results align with those reported by Feizpour et al. [28], who validated the Lumipulse G pTau_217_ assay against PET imaging, showing strong discrimination between A + and A − individuals (AUC 0.91–0.94). While their study relied on PET as a reference, our study compared pTau_217_ to CSF biomarkers, which are more widely used in clinical practice. Similarly, Cecchetti et al. [41] reported high diagnostic accuracy (AUC > 0.95) for plasma pTau_217_ in a prospective cohort, consistent with our findings.

Regarding storage conditions, pTau_217_ stability was evaluated by comparing the effect of freezing plasma samples within 4 h compared to keeping them refrigerated for 18 h before freezing. Results indicated no significant differences in pTau_217_ concentrations between both conditions, suggesting that pTau_217_ remains stable for up to 18 h under refrigeration. This finding supports the feasibility of transporting samples to external laboratories for analysis, benefiting centers without in-house testing capabilities, and thus expanding its accessibility. Consistent with our findings, a recent study using Liquid Chromatography–tandem Mass Spectrometry (LC–MS/MS) also demonstrated pTau_217_ stability following delayed centrifugation and storage [42].

Analytical variability is another crucial factor in the practical implementation of a biomarker. It is important to recognize that data fluctuations arised from lot-to-lot differences could have a significant impact on the stability of cutoffs. However, no significant variability was observed in our study indicating consistency in pTau_217_ measurements across different assay lots and ensuring that results were attributable to the presence or absence of AD pathophysiology and not to analytical variability. On the other hand, when assessing potential variations in pTau_217_ concentrations across different laboratory protocols (clinical versus research), we observed slight but significant differences, highlighting the importance of evaluating such differences prior to its implementation in clinical practice. Collectively, these findings provide valuable insights into the sample collection, storage, and analysis of pTau_217_ using an automated platform, representing essential knowledge to support its implementation in a clinical setting.

We evaluated the generalizability of our findings to participants without a reference standard. In our cohort, we observed biomarker differences between individuals eligible for CSF analysis and those who underwent blood-only testing. Four key factors, age, clinical stage, chronic kidney disease and the inclusion in the clinical protocol, may explain these differences. Patients who did not undergo lumbar puncture were generally older, with more comorbidities and in more advanced clinical stages, with an expected higher prevalence of amyloid pathology. These findings underscore the importance of considering the context of use when interpreting biomarker results [43]. This is particularly relevant for blood-based biomarkers, as patient selection criteria in clinical practice may differ from those in CSF-based studies. The effect of renal dysfunction on plasma pTau_217_ concentrations has been previously reported [44]. The effect of age and clinical syndrome on the accuracy of plasma pTau_217_ has been further investigated in other platforms. Therriault et al. [45] demonstrated that in patients with mild cognitive impairment, the PPV of pTau_217_ increased with age, exceeding 95% in patients aged 90 +, while the NPV declined in older individuals due to the higher prevalence of Aβ pathology. In our study we found the same behavior of the biomarker in different prevalence scenarios, with the corresponding implications for the interpretation of its results.

The main strength of our study lies in its reflection of the real-world context of a memory clinic. By incorporating the prospective measurement of plasma pTau_217_ into our workflow alongside the clinical laboratory, we demonstrated its feasibility for integration into our diagnostic clinical routine. On the other hand, the analysis of paired blood samples under varying pre-analytical conditions allowed us to confirm the stability of the biomarker, ensuring its reliability across different handling processes. Additionally, leveraging the prospective design, we examined the variability across multiple assay lots to assess consistency in measurements, a critical factor for its successful implementation in clinical workflows. These methodological strengths enhance the robustness and applicability of our results. But our study has also some limitations. On the one hand, not all participants had CSF samples available as a reference and those who did tended to be slightly younger and at earlier disease stages, introducing a potential bias. On the other hand, as the study was conducted at a single specialized memory clinic, the results reflect this context but should be validated in other settings where the target population may have different characteristics. Another limitation is that the pTau217 concentration in the low control used for lot-to-lot comparisons (0.49 pg/mL) is closer to the average levels seen in A + individuals than in A- individuals. Lastly, the validation has been performed against CSF and not against PET or neuropathology.

## Conclusion

The implementation of plasma biomarkers for identifying AD has important implications in clinical practice, including simplified logistics and resources in the diagnostic process. The stability under different storage conditions, the low lot-to-lot variability of plasma pTau_217_ measurements in an automated platform, and the robustness of its accuracy under different protocol measurements result in high diagnostic performance. However, to maximize its clinical utility, when using the two-threshold approach, it is essential to optimize the trade-off between diagnostic accuracy and the proportion of individuals classified within the gray zone, ensuring a balance between precision and practical applicability. Additionally, results must be interpreted within the specific clinical setting, as factors like disease prevalence can influence biomarker performance. Altogether, our findings support the integration of plasma pTau_217_ into routine clinical practice in memory clinics, providing clinicians with a reliable biomarker for AD diagnosis and facilitating the selection of patients for disease-modifying treatments.

## Supplementary Information

Supplementary Material 1.

## Data Availability

Raw anonymized data and code for statistical analysis are available upon reasonable request. All requests should be sent to the corresponding author detailing the study hypothesis and statistical analysis plan. The steering committee of this study will decide whether data/code sharing is appropriate based on the novelty and scientific rigor of the proposal. All applicants will be asked to sign a data access agreement.
