# Supplementary material for: Prospective evaluation of plasma pTau217 stability for the detection of Alzheimer’s disease in a tertiary memory clinic
Source: Alzheimers Res Ther. 2025 Jul 5;17:150. doi: 10.1186/s13195-025-01779-7 (PMC12228402; doi:10.1186/s13195-025-01779-7)
Supplement: Supplementary file 1 — Supplementary Material 1. [file 13195_2025_1779_MOESM1_ESM.docx]

**Supplementary Material**

**Methods:**

**Supplementary Text 1:** **Participants with intermediate concentrations of pTau217.**

With strict cutoffs applied, individuals in the grey zone had a mean age of 75 years. Within this group, no differences were observed in sex distribution (54% female, 46% male, p-value = 0.35) or amyloid positivity in participants with CSF available (42% A+, 58% A-, p-value = 0.26). Over 70% were in MCI stage, and 18% of the participants had eGFR <60 mL/min/m2 (**Supplementary Table 1**)

Applying lenient cutoffs, individuals in the grey zone had a mean age of 75 years, with no observed differences in sex distribution (59% female, 41% male, p-value = 0.15). 75% of individuals were A+ in participants with CSF available (p=0.025) and most participants were in the MCI stage (>75%). The prevalence of eGFR < 60 mL/min/m2 was 19% (**Supplementary Table 2**).

**Results**

**Supplementary Table 1. Characteristics of patients stratifying by historical strict cutoffs**

| Characteristic | < 0.13  N = 80 | 0.13 - 0.55  N = 137 | > 0.55  N = 63 | p-value |
| --- | --- | --- | --- | --- |
| Age (years) | 69 (8) | 75 (7) | 76 (7) | <0.001 |
| Sex (Female) | 41 (51%) | 74 (54%) | 31 (49%) | 0.8 |
| Plasma pTau217 (pg/mL) | 0.09 (0.02) | 0.27 (0.13) | 1.08 (0.71) | <0.001 |
| Plasma Aβ_1-42_/Aβ_1-40_ | 0.076 (0.006) | 0.074 (0.014) | 0.070 (0.007) | 0.3 |
| CSF Amyloid positive status | 2 (5.3%) | 29 (58%) | 21 (100%) | <0.001 |
| GDS | |  |  | <0.001 |
| GDS <= 3 | 62 (79%) | 95 (71%) | 25 (40%) |  |
| GDS >= 4 | 16 (21%) | 38 (29%) | 37 (60%) |  |
| eGFR | |  |  | <0.001 |
| > 90 | 37 (46%) | 16 (12%) | 4 (6.3%) |  |
| 60-90 | 40 (50%) | 92 (67%) | 42 (67%) |  |
| <60 | 3 (3.8%) | 29 (21%) | 17 (27%) |  |
| CSF availability | |  |  | 0.2 |
| With CSF | 38 (48%) | 50 (36%) | 21 (33%) |  |
| Without CSF | 42 (53%) | 87 (64%) | 42 (67%) |  |
| Clinical diagnosis | |  |  |  |
| Alzheimer's Disease | 3 (3.8%) | 63 (46%) | 52 (83%) |  |
| Frontotemporal Lobal Degeneration | 17 (21%) | 14 (10%) | 4 (6.3%) |  |
| Lewy Body Disease | 13 (16%) | 24 (18%) | 4 (6.3%) |  |
| Other - Non-Neurodegenerative | 42 (53%) | 33 (24%) | 3 (4.8%) |  |
| Unknown | 5 (6.3%) | 3 (2.1%) | 0 (0%) |  |

​**Supplementary Table 2. Characteristics of patients stratifying by historical lenient cutoffs**

| Characteristic | < 0.19  N = 128 | 0.19 - 0.39  N = 59 | > 0.39  N = 93 | p-value |
| --- | --- | --- | --- | --- |
| Age (years) | 70 (8) | 75 (7) | 77 (6) | <0.001 |
| Sex (Female) | 64 (50%) | 35 (59%) | 47 (51%) | 0.5 |
| Plasma pTau217 (pg/mL) | 0.11 (0.04) | 0.26 (0.06) | 0.88 (0.65) | <0.001 |
| Plasma Aβ_1-42_/Aβ_1-40_ | 0.079 (0.010) | 0.071 (0.016) | 0.070 (0.009) | 0.024 |
| CSF Amyloid positive status | 7 (12%) | 15 (75%) | 30 (100%) | <0.001 |
| GDS | |  |  | <0.001 |
| GDS <= 3 | 98 (78%) | 43 (75%) | 41 (45%) |  |
| GDS >= 4 | 27 (22%) | 14 (25%) | 50 (55%) |  |
| eGFR | |  |  | <0.001 |
| > 90 | 43 (34%) | 9 (15%) | 5 (5.4%) |  |
| 60-90 | 72 (56%) | 39 (66%) | 63 (68%) |  |
| <60 | 13 (10%) | 11 (19%) | 25 (27%) |  |
| CSF availability | |  |  | 0.077 |
| With CSF | 59 (46%) | 20 (34%) | 30 (32%) |  |
| Without CSF | 69 (54%) | 39 (66%) | 63 (68%) |  |
| Clinical diagnosis | |  |  |  |
| Alzheimer's Disease | 10 (7.8%) | 30 (51%) | 78 (84%) |  |
| Frontotemporal Lobal Degeneration | 27 (21%) | 3 (5.1%) | 5 (5.4%) |  |
| Lewy Body Disease | 26 (20%) | 9 (15%) | 6 (6.5%) |  |
| Other - Non-Neurodegenerative | 59 (46%) | 16 (27%) | 3 (3.2%) |  |
| Unknown | 6 (4.7%) | 1 (1.7%) | 1 (1.1%) |  |

**Supplementary Table 3. Analytical variability; lot-to-lot variability.**

| Run | Day of analysis | Lot | Control N1 (pg/mL) | Control N2 (pg/mL) |
| --- | --- | --- | --- | --- |
| 1 | Day 1 | 4097 | 0.446 | 3.786 |
| 2 | Day 1 | 4097 |  | 3.889 |
| 3 | Day 2 | 4097 | 0.477 | 3.951 |
| 4 | Day 3 | 4097 | 0.489 | 3.887 |
| 5 | Day 4 | 4097 | 0.497 | 3.863 |
| 6 | Day 5 | 4097 | 0.48 | 3.833 |
| 7 | Day 6 | 4097 | 0.488 | 3.986 |
| 8 | Day 7 | 4129 |  | 3.81 |
| 9 | Day 8 | 4129 | 0.461 |  |
| 10 | Day 9 | 4097 | 0.492 | 3.951 |
| 11 | Day 9 | 4097 | 0.498 | 4.044 |
| 12 | Day 9 | 4129 | 0.499 | 3.964 |
| 13 | Day 10 | 5023 | 0.5 | 4.013 |
| 14 | Day 11 | 4129 |  | 3.879 |
| 15 | Day 12 | 4129 |  | 3.918 |
| 16 | Day 13 | 4129 | 0.488 |  |
| 17 | Day 13 | 5023 | 0.504 |  |
| 18 | Day 14 | 5023 |  | 3.813 |
| 19 | Day 15 | 5023 |  | 3.899 |
| 20 | Day 16 | 5023 | 0.461 |  |
| 21 | Day 17 | 4129 | 0.484 |  |
| 22 | Day 17 | 4129 | 0.502 |  |
| 23 | Day 18 | 4129 | 0.497 |  |
| 24 | Day 19 | 5023 | 0.481 | 3.884 |
| 25 | Day 20 | 4129 |  | 3.896 |
| 26 | Day 21 | 4129 | 0.481 | 3.895 |

In Supplementary Table 3 we show the number of runs performed and their date, the lots of reagents, and the values of Control N1 (low control) and Control N2 (high control).

**Supplementary Figure 1. Diagnostic accuracy of plasma pTau217 for the discrimination of A positive from A negative categories**


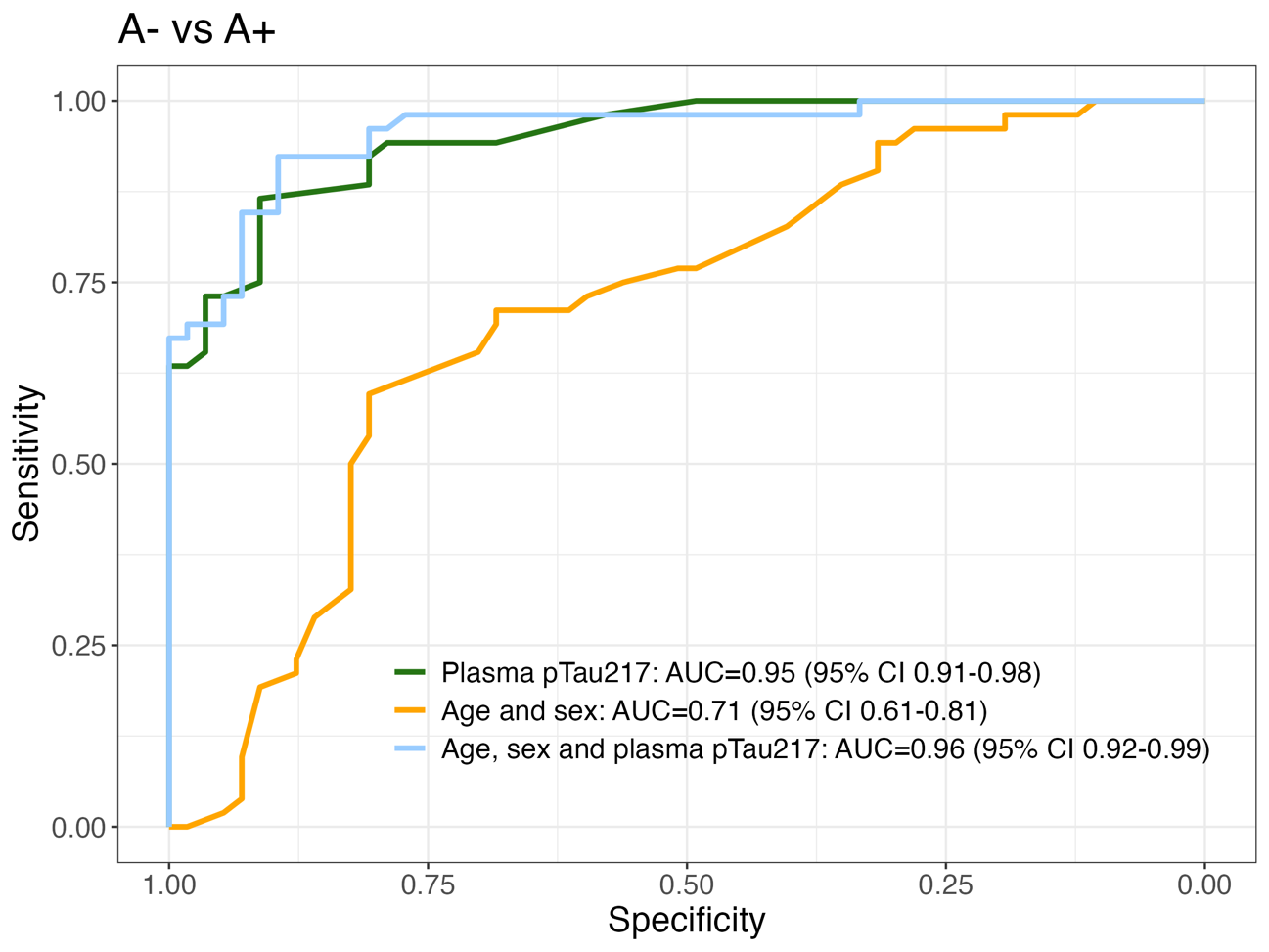


AUROC of plasma biomarkers and their combinations. Plasma pTau_217_ reached an AUC of 0.95 without being outperformed by any other combination (DeLong test).

**Supplementary Figure 2.** **Variations in PPV, NPV and global accuracy of the different cutoff combinations in different clinical scenarios with different prevalence of amyloid pathology**

The figure illustrates how low prevalences of amyloid pathology reduce the PPV, whereas high prevalences decrease the NPV.

Abbreviations: PPV, positive predictive value. NPV, negative predictive value.
